# DNN-Based Estimation for Misalignment State of Automotive Radar Sensor

**DOI:** 10.3390/s23146472

**Published:** 2023-07-17

**Authors:** Junho Kim, Taewon Jeong, Seongwook Lee

**Affiliations:** 1 School of Electrical and Electronics Engineering, College of ICT Engineering, Chung-Ang University, 84 Heukseok-ro, Dongjak-gu, Seoul 06974, Republic of Korea; keemjuno@cau.ac.kr; 2 School of Electronics and Information Engineering, College of Engineering, Korea Aerospace University, 76 Hanggongdaehak-ro, Deogyang-gu, Goyang-si 10540, Republic of Korea; wjdxoone@kau.kr

**Keywords:** automotive radar, deep neural network, frequency-modulated continuous wave radar, misalignment, tilt angle

## Abstract

The reliability and safety of advanced driver assistance systems and autonomous vehicles are highly dependent on the accuracy of automotive sensors such as radar, lidar, and camera. However, these sensors can be misaligned compared to the initial installation state due to external shocks, and it can cause deterioration of their performance. In the case of the radar sensor, when the mounting angle is distorted and the sensor tilt toward the ground or sky, the sensing performance deteriorates significantly. Therefore, to guarantee stable detection performance of the sensors and driver safety, a method for determining the misalignment of these sensors is required. In this paper, we propose a method for estimating the vertical tilt angle of the radar sensor using a deep neural network (DNN) classifier. Using the proposed method, the mounting state of the radar can be easily estimated without physically removing the bumper. First, to identify the characteristics of the received signal according to the radar misalignment states, radar data are obtained at various tilt angles and distances. Then, we extract range profiles from the received signals and design a DNN-based estimator using the profiles as input. The proposed angle estimator determines the tilt angle of the radar sensor regardless of the measured distance. The average estimation accuracy of the proposed DNN-based classifier is over 99.08%. Therefore, through the proposed method of indirectly determining the radar misalignment, maintenance of the vehicle radar sensor can be easily performed.

## 1. Introduction

With the growing interest in autonomous driving systems, demand for automotive sensors, such as radar, lidar, and camera, is also increasing [1]. Radar, short for radio detection and ranging, employs electromagnetic waves to detect targets and estimate the range and velocity information of targets [2]. The radar system transmits the electromagnetic waves via transmitting antenna elements and receives the returning echo signal from the target through receiving antenna elements. This echo signal is then processed to extract the information about the target, such as its range and velocity. Similarly, lidar, an acronym for light detection and ranging, is a sensor that uses pulsed laser light to estimate the range of the targets [3]. While it shares the capability with radar to determine the range and velocity of the targets, lidar distinguishes itself by using light waves instead of electromagnetic waves.

These sensors provide various functions for driver assistance systems and driver safety, including advanced cruise control, collision damage mitigation, and pre-crash safety [4,5,6,7,8]. For the effective functioning of these sensors, a proper calibration process is required. In general, parameters to be considered for calibration are divided into intrinsic and extrinsic calibration parameters [9,10,11]. For radar sensors, intrinsic calibration includes the parameters needed for precise determination of the target’s range, velocity, and angle of arrival. These parameters can involve such as the distance between antennas, phase and gain mismatch, and characteristics of the chirp signal. On the other hand, the extrinsic calibration parameters include the radar’s translation and rotation parameters, which specify the radar mounting position and angle from the initially mounted reference point [12,13].

In the case of automotive sensor, the initial alignment may be distorted due to various external shocks and continuous vibration of the vehicle while driving [14]. In particular, because the radar sensor detects targets using radio waves if the vertical mounting angle is tilted and the transmitted signal is directed toward the ground or sky, sensing performance can be degraded [15]. Therefore, maintaining the initial alignment condition of the radar sensor is essential to guarantee the stable detection performance of the radar. In general, to discriminate the mounting state of the automotive radar sensor, the bumper should be removed to check the mounting state directly, which involves a lengthy process and incurs high expenses. Thus, an indirect method of determining the radar mounting state and estimating the radar misalignment angle is needed. Therefore, in this paper, we propose a method to indirectly determine the misalignment of vehicle radar for ease of post-vehicle maintenance.

Several studies have been conducted to indirectly estimate the misalignment of various automotive sensors. In [16], authors proposed a method to estimate the misalignment of lidar by applying a nonlinear least square optimization method with the Levenberg-Marquardt algorithm. The authors in [17] estimated the yaw misalignment of an inertial measurement unit in a vehicle using onboard sensors and the Kalman filter. Also, in [18], the authors employ the one-dimension Kalman filtering method to estimate the correction value for each sector individually. Subsequently, this correction value was utilized to rectify the horizontal misalignment of the radar sensor. Moreover, in the case of the radar sensor, the authors in [19] proposed a method of estimating the angle of horizontal misalignment of the radar through a stationary target while driving the vehicle. The authors in [20] also estimate the angle of horizontal misalignment through a straight stationary reference (i.e., guardrail). On the other hand, the authors in [21] estimate the vertical misalignment angle by using the received signal of the radar. They extracted features representing the statistical characteristics of the distribution, such as the mean, variance, variance coefficient, kurtosis, skewness, and maximum value of the received signal, and then applied the principal component analysis [22] and the *k*-nearest neighbors algorithm [23].

In addition, studies applying deep learning in the field of radar signal processing are being actively conducted [24]. In automotive radar signal processing, deep learning is mainly applied for target detection or classification [25,26,27,28,29]. The authors in [26] use range-Doppler maps as input of the deep learning network for target detection. For target classification, images of the micro-Doppler spectrogram were used as input of the DNN in [27] and authors in [28] use both range-Doppler map and Doppler-time map as input. Moreover, it can be applied in a variety of ways in the radar signal processing, such as mitigating interference [30] or estimating the angle of arrival [31].

In this paper, we propose a deep neural network (DNN)-based classifier to estimate the vertical misalignment angle of the frequency-modulated continuous wave (FMCW) radar sensor by measuring the signal strength of a specific target at a specific distance. First, we obtain radar data according to various vertical tilt angles (e.g., $-45^{\circ },\;-30^{\circ },\;-15^{\circ },\;0^{\circ },\;15^{\circ },\;30^{\circ },\;45^{\circ }$) while changing the measurement distances. Then, range profiles are extracted from the acquired radar sensor data and they are used as inputs for training the DNN-based classifier. In the end, by adjusting the number of hidden layers in the DNN and the nodes used in each layer, we select a structure that shows the highest accuracy with minimal computation for our proposed misalignment angle estimator.

In summary, the major contributions of our work can be summarized as follows:

- The misalignment angle of the radar sensor can be estimated through the proposed DNN-based classifier.
- Unlike previous methods that directly check the radar mounting state by overhauling the bumper, the proposed method can indirectly estimate the radar misalignment angle.
- Compared with [21], the proposed DNN-based estimation method does not specifically require the step of selecting appropriate features.
- The proposed DNN-based estimation method shows higher accuracy than feature-based machine learning methods such as decision tree.

**Figure 1:**
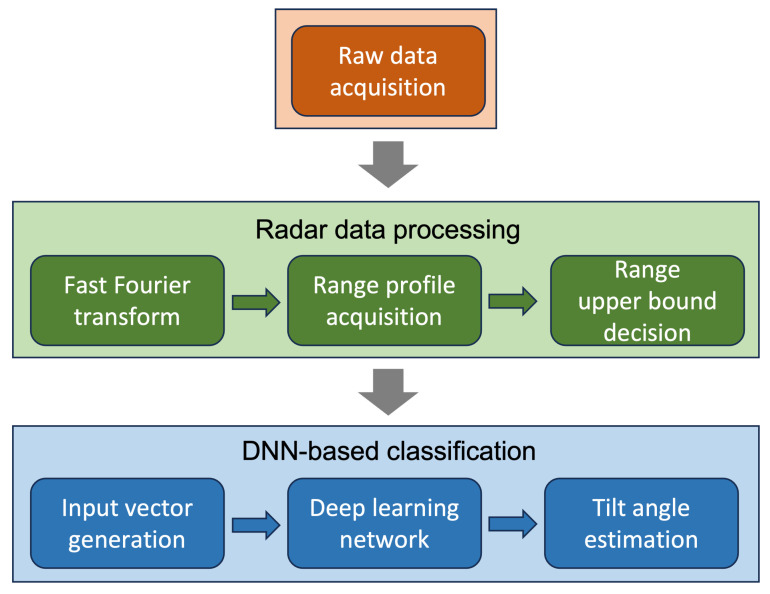
Holistic pipeline of proposed method to estimate misalignment state.

The remainder of this paper is organized as follows. In Section 2, we describe the basic principles of the FMCW radar system. Then, in Section 3, the measurement environment and the characteristics of range profiles acquired in the environment are described. In Section 4, a DNN-based classifier for estimating the tilt angle is proposed and also its estimation performance is evaluated. Finally, concluding summaries are drawn in Section 5. The holistic pipeline of our proposed method is shown in Figure 1.

## 2. Signal Model in FMCW Radar System

**Figure 2:**
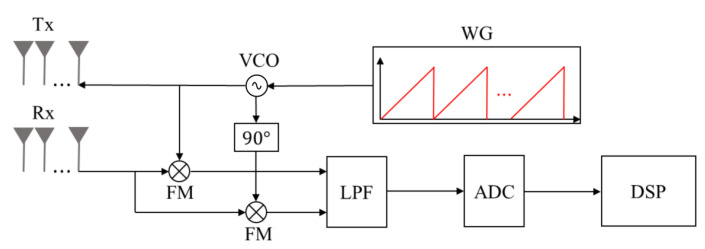
General block diagram of the FMCW radar system.

**Figure 3:**
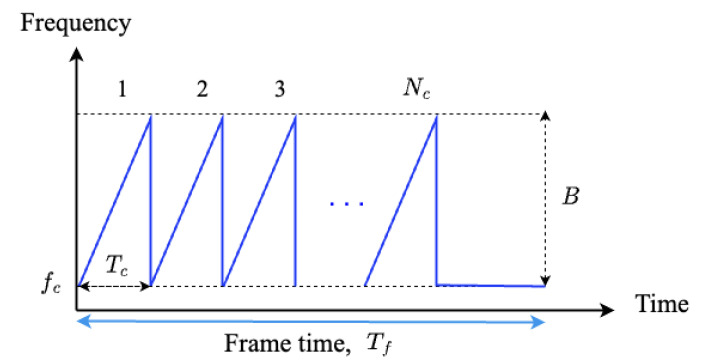
Transmitted signal in the FMCW radar system: form of repetitive chirp signal [33].

The FMCW radar system, which is mainly used in automotive radar systems, consists of transmit antenna elements (Tx), receiving antenna elements (Rx), waveform generator (WG), voltage-controlled oscillator (VCO), frequency mixer (FM), low-pass filter (LPF), analog-to-digital converter (ADC), and digital signal processor (DSP), as shown in Figure 2 [32]. The waveform generator generates a transmission signal with a frequency that linearly changes over time as shown in Figure 3. The *i*-th (*i* = 1, 2, …, $N_{c}$) chirp in the transmitted waveform can be expressed in time-domain as

(1) $$\begin{array}{c} s_{i}\left(t\right)=A_{i}\cos\left(2\pi f_{c}t+\pi \frac{B}{T_{c}}t^{2}\right)\;\left(\left(n-1\right)T_{c}\leq t\leq nT_{c}\right), \end{array}$$

where $A_{i}$, $f_{c}$, *B*, and $T_{c}$ represent the amplitude, center frequency, bandwidth, and duration of each chirp, respectively.

Then, the sum of received signals reflected from the *k*-th target (k=1,2,…,K) is expressed as follows:

(2) $$\begin{array}{cc} s_{r}\left(t\right)= & \sum_{k=1}^{K}A_{r,\;k}\cos\left\{2\pi \left(f_{c}+f_{d,\;k}\right)\left(t-t_{0,\;k}\right)+\pi \frac{B}{T_{c}}{\left(t-t_{0,\;k}\right)}^{2}\right\}\\ \; & (\left(n-1\right)T_{c}+t_{0,\;k}\leq t\leq nT_{c}), \end{array}$$

where $A_{r,\;k}$ represents the received signal strength. In addition, $f_{d,\;k}=\frac{2V_{k}f_{c}}{c}$ is the Doppler frequency caused by the velocity of the *k*-th target and $t_{0,\;k}=\frac{2R_{k}}{c}$ is the time delay caused by the distance between the *k*-th target and the radar, where $R_{k}$, $V_{k}$, and *c* denote the distance to the *k*-th target, velocity of the *k*-th target, and the speed of light, respectively.

Then, the received signal is passed through a frequency mixer along with the transmitted signal. After passing through a low-pass filter, the output of the mixer yields an in-phase (I) baseband signal, which can be expressed as

(3) $$\begin{array}{cc} s_{d,\;I}\left(t\right)=\sum_{k=1}^{K} & \frac{A_{i}A_{r,\;k}}{2}\cos\left\{2\pi \left(\frac{B}{T_{c}}t_{0,\;k}-f_{d,\;k}\right)t+2\pi t_{0,\;k}f_{c}\right\}\;\left(\left(n-1\right)T_{c}+t_{0,\;k}\leq t\leq nT_{c}\right). \end{array}$$

The signal of (3) is passed through an analog-to-digital converter and the discrete-time signal can be expressed as follows:

(4) $$\begin{array}{cc} s_{d,\;I}\left[n\right]= & \sum_{k=1}^{K}\frac{A_{i}A_{r,\;k}}{2}\cos\left\{2\pi \left(\frac{B}{T_{c}}t_{0,\;k}-f_{d,\;k}\right)nT_{s}+2\pi t_{0,\;k}f_{c}\right\}\\ \; & (n=0,\;1,\;\ldots ,\;N-1), \end{array}$$

where $T_{s}$ is the sampling period and *N* is the number of time samples. In addition, a quadrature (Q) baseband signal can be obtained by applying the same process with a $90^{\circ }$ phase-shifted transmitted signal, which can be expressed as

(5) $$\begin{array}{cc} s_{d,\;Q}\left[n\right]= & \sum_{k=1}^{K}\frac{A_{i}A_{r,\;k}}{2}\sin\left\{2\pi \left(\frac{B}{T_{c}}t_{0,\;k}-f_{d,\;k}\right)nT_{s}+2\pi t_{0,\;k}f_{c}\right\}\\ \; & (n=0,\;1,\;\ldots ,\;N-1). \end{array}$$

The final IQ baseband signal can be obtained by combining the two signals of (4) and (5), which can be expressed as

(6) $$\begin{array}{cc} s_{d}\left[n\right]= & s_{d,\;I}\left[n\right]+js_{d,\;Q}\left[n\right]\\ = & \sum_{k=1}^{K}\frac{A_{t}A_{r,\;k}}{2}\exp\left[j2\pi \left\{\left(\frac{B}{T_{c}}t_{0,\;k}-f_{d,\;k}\right)nT_{s}+t_{0,\;k}f_{c}\right\}\right]\\ \; & (n=0,\;1,\;\ldots ,\;N-1). \end{array}$$

The distance to the target can be estimated from the frequency of (6) [33]. Thus, the fast Fourier transform (FFT) is applied to extract the frequency and the frequency-domain baseband signal can be expressed as

(7) $$\begin{array}{cc} S_{d}\left[m\right]= & \sum_{n=0}^{N-1}s_{d}\left[n\right]\exp\left(\frac{-j2\pi mn}{N}\right)\;\left(m=0,\;1,\;\ldots ,\;N-1\right), \end{array}$$

where *m* indicates the frequency index in the frequency-domain. The magnitude of the baseband signal in the frequency-domain is defined by the range profile. In general, the time delay of wave propagation due to the distance between the radar and the target (i.e., $\frac{B}{T_{c}}t_{0,k}$) is much larger than the Doppler shift (i.e., $f_{d,k}$). Therefore, the Doppler shift can be ignored. When the frequency corresponding to the peak value from the result of the FFT is denoted as $\hat{f_{k}}$, the distance between the radar and the target can be obtained as follows:

(8) $$R_{k}=\hat{f_{k}}\times \frac{T_{c}c}{2B}.$$

## 3. Measurement and Analysis for FMCW Radar Sensor Data

In this section, we describe our experimental setup and analyze the obtained radar data. First, we describe the specifications of the radar system and our experimental environment. Then, we analyze how the received radar signals are affected by the tilt angle in the range profile.

### 3.1. Measurement Environment

**Figure 4:**
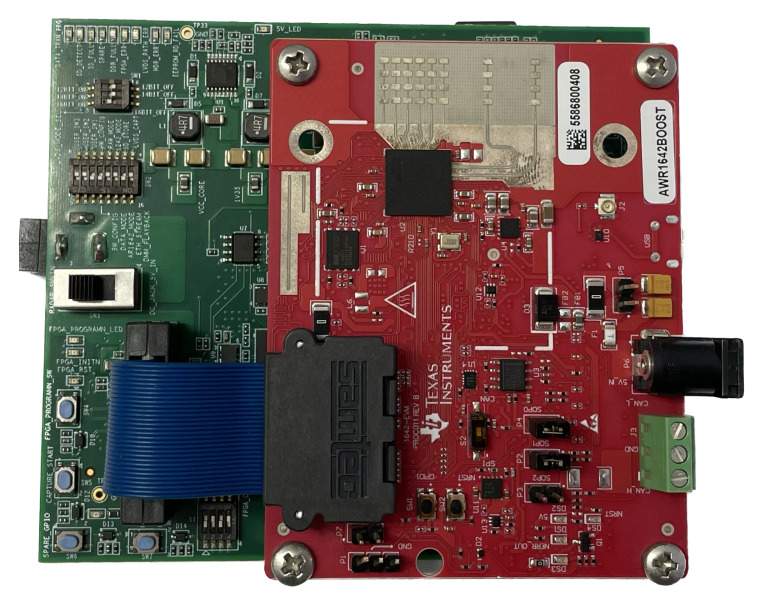
AWR1642BOOST and DCA1000EVM manufactured by Texas Instruments.

In our measurements, we obtained radar data using an AWR1642BOOST board manufactured by Texas Instruments [34]. We used the AWR1642BOOST board connected with a DCA1000EVM, as shown in Figure 4. The radar sensor uses a center frequency and a bandwidth of 77 GHz and 3 GHz, respectively. In addition, a total of 128 chirps are used and 256 time samples are taken from each chirp. Also, the chirp duration is 160 μs and the frame time corresponding to one signal processing cycle is 20.48 ms. In this measurement environment, 1 transmit antenna and 4 receiving antenna elements are used. The specifications of the radar used in the measurement are summarized in Table 1.

**Figure 5:**
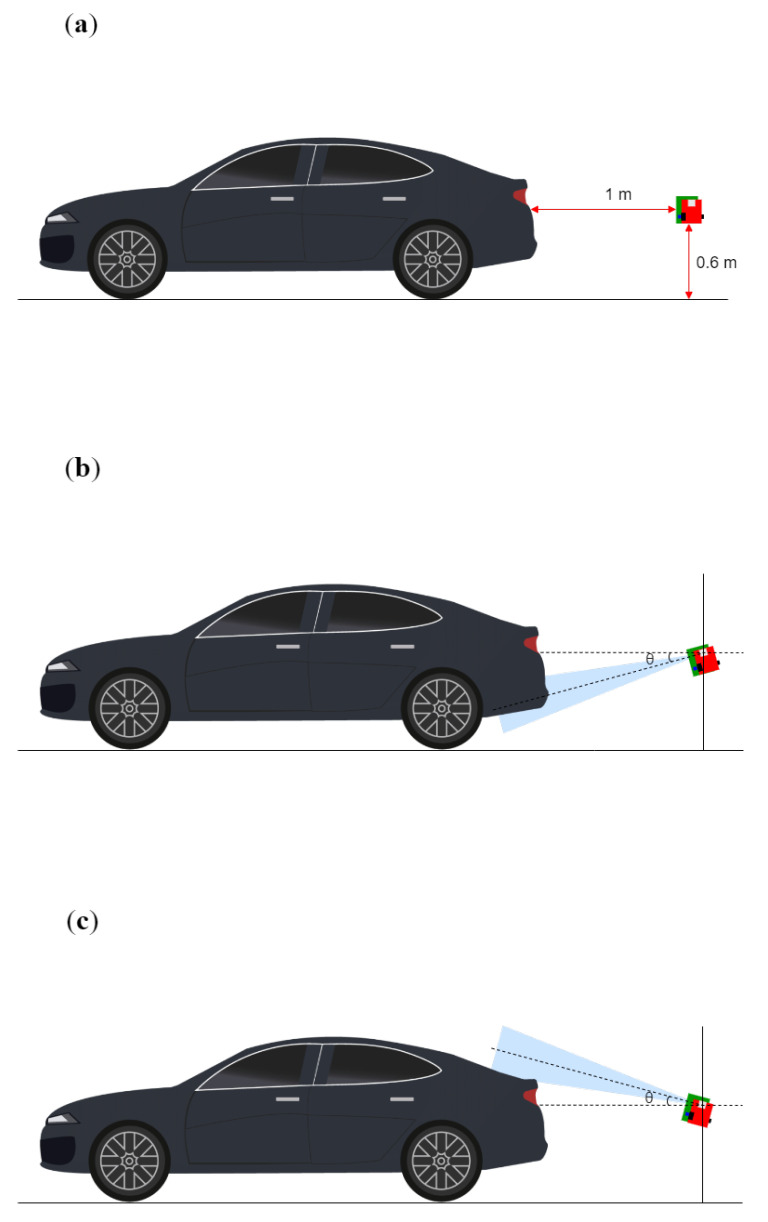
Examples for radar signal measurements: (**a**) 0${}^{\circ }$ (reference angle), (**b**) tilted at a negative angle, and (**c**) tilted at a positive angle.

With this radar system, we obtained the radar sensor data from various tilt angles while changing the measurement distances, which is shown in Figure 5. As shown in Figure 5, the radar sensor is positioned behind the target, and the sensor data are obtained by adjusting the tilt angle of the radar. Furthermore, the radar sensor is positioned 0.6 m above the ground, corresponding to the typical installation height of an automotive radar sensor at the vehicle bumper. Then, the sensor data are measured according to elevation angles ranging from $-45^{\circ }$ to $45^{\circ }$ at measurement distances of 1 m, 2 m, and 3 m between the target and the radar. The angular interval is set to $15^{\circ }$ to obtain data for 7 different tilt angles for each measurement distance.

A steel trihedral corner reflector with a side length of 20 cm and two vehicles are used as targets. First, the range profiles according to the measurement angles and distances are obtained using a corner reflector. Based on the acquired corner reflector data, the input of the proposed vertical tilt angle estimator is determined and the structure of the DNN is designed. Then, the measurement results on the actual vehicles are used for training and verification of the proposed DNN along with corner reflector data. Measurement scenarios are summarized in Table 2.

### 3.2. Analysis of Range Profiles

**Figure 6:**
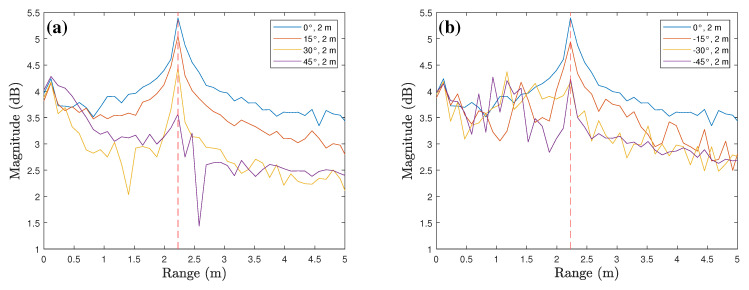
Range profiles according to various tilt angles: (**a**) a radar mounted at positive angles and (**b**) a radar mounted at negative angles.

**Figure 7:**
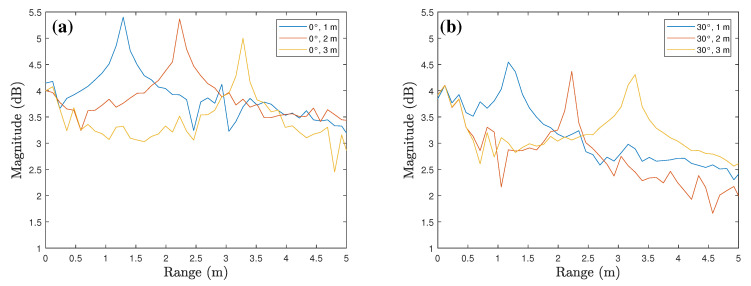
Range profiles according to the measurement distances: the tilt angles are (**a**) 0${}^{\circ }$ and (**b**) 30${}^{\circ }$.

The range profiles according to various tilt angles for corner reflector are shown in Figure 6. Figure 6a shows the comparison of range profiles of $0^{\circ },15^{\circ },30^{\circ }$, and $45^{\circ }$ tilt angles when the measurement distance is 2 m. As shown in the figure, the peak values occur around 2 m where the target is located. Moreover, there is a gradual decrease in the average magnitude of the range profile as the tilt angle increase. Figure 6b shows the range profiles for negative tilt angles, and even in this case, the peak values occur around 2 m and the average magnitude decreases as the angle deviates from $0^{\circ }$. In addition, Figure 7 shows the comparison of range profiles according to the measurement distances. In both cases where the tilt angles are $0^{\circ }$ and $30^{\circ }$, the peak values gradually decrease as the measurement distance increases from 1 m to 3 m.

## 4. Proposed DNN-Based Tilt Angle Estimation Method

### 4.1. Input Vector Generation for Training

As mentioned in Section 3.2, the range profile includes the signal strength information according to the radar tilt at each measurement distance. Thus, we propose to use the range profile as an input for the DNN-based tilt angle estimator. Because we detect a target at a certain distance and discriminate the misalignment angle of the radar sensor, using the full-range profile as the input vector of the DNN may reduce efficiency. Therefore, for efficient tilt angle estimation, it is necessary to extract the input vector including the signal component for the target instead of the entire range.

**Figure 8:**
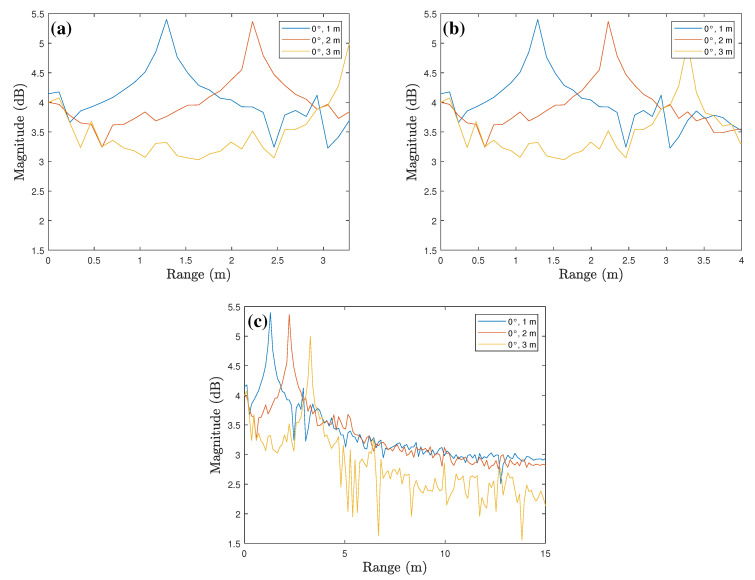
Range profiles for the input of the proposed DNN-based classifier: between (**a**) 0 m and peak of the furthest target, (**b**) 0 and 4 m, and (**c**) 0 and 15 m.

Figure 8 represents the range profiles including the signal component of the target. Figure 8a shows the range profile cropped based on the measured distance of the target around 3 m. In this case, the signal component representing a detection result for the target around 3 m is not fully included and is cut off based on the peak. Figure 8b shows a range profile between 0 and 4 m, where the signal components representing the detection result for all three targets are presented. Figure 8c, which shows a range profile between 0 and 15 m, also includes the signal components representing the detection result for all three targets, but in this case, many unnecessary signal components are included. Thus, for efficient radar sensor misalignment angle estimation, it is appropriate to use a range profile between 0 and 4 m as shown in Figure 8b. As shown in Table 1, the range resolution in our measurement environment is 0.1172 m, so the total length of the input vectors is set to 34 (i.e., 0.1172 × 34 = 3.9848).

After that, we define the training and test datasets to be used in training the proposed DNN-based classifier and validating its performance. In our measurements, 100 frames of data were obtained with 4 different channels. The range profiles are not exactly identical due to the phase difference that exists between each channel (i.e., $N_{T}$×$N_{R}$). Thus, the data set consisting of a total of 400 range profiles was obtained for each specific tilt angle and measured distance. As a result, the data set consisting of a total of 8400 range profiles were generated with 7 tilt angles and 3 measurement distances for each target. Finally, we split the training and test data into 7200 and 1200 range profiles.

### 4.2. Proposed DNN-based Misalignment Angle Estimator

In this section, we design a DNN-based classifier for estimating the misalignment angles from $-45^{\circ }$ to $45^{\circ }$ with $15^{\circ }$ intervals based on the vectors extracted from the range profiles. In other words, the proposed DNN outputs one value out of seven angles (i.e., $-45^{\circ }$, $-30^{\circ }$, $-15^{\circ }$, $0^{\circ }$, $15^{\circ }$, $30^{\circ }$, and $45^{\circ }$) regardless of measured distance when the input vector passes through the proposed DNN. In general, the DNN for classification consists of an input layer, hidden layers, an output layer, and a softmax layer [35]. We used a total of 34 nodes in the input layer to match the size of the input vector. In addition, the output layer consists of nodes corresponding to 7 tilt angles, and the softmax layer converts the values calculated at the output layer into the form of probability. As a result, the highest probability value represents the estimated tilt angle for the corresponding input vector.

**Figure 9:**
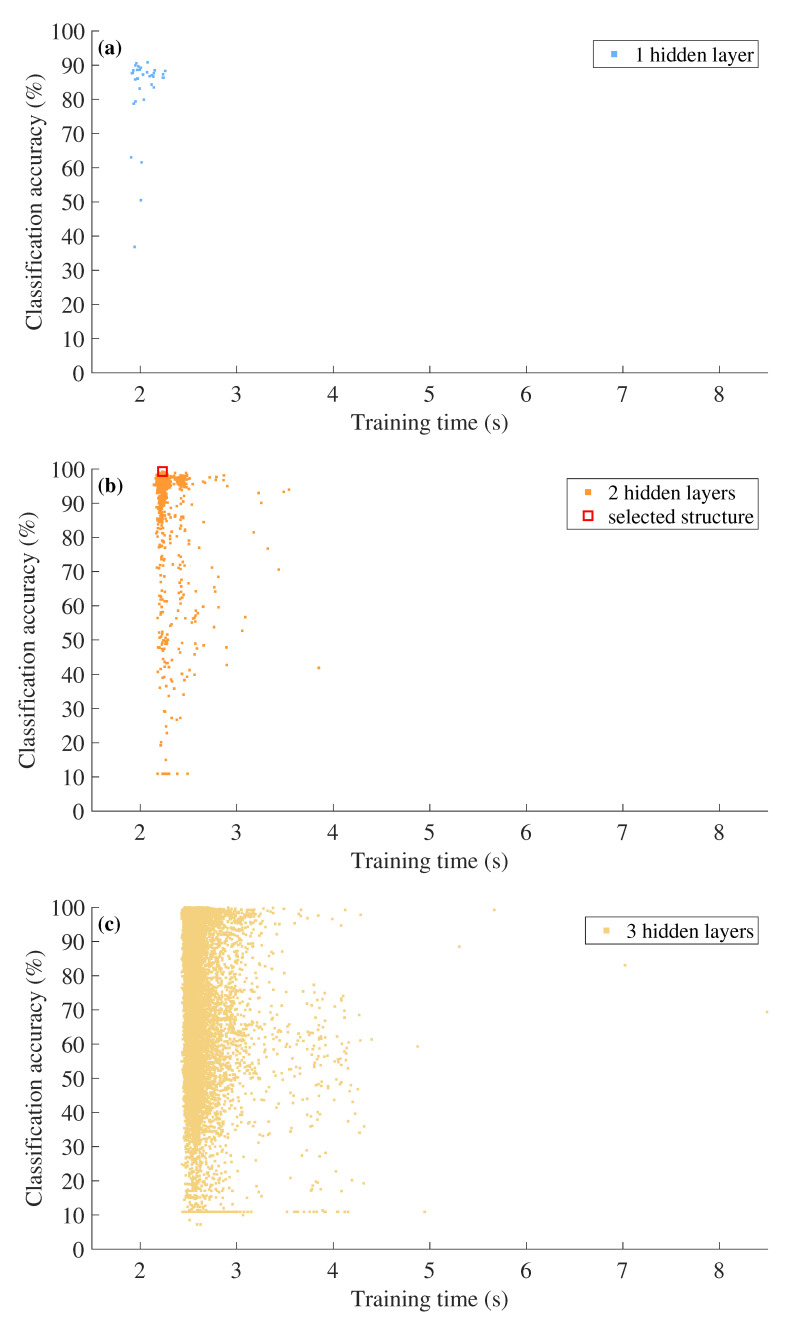
Estimation accuracy and training time in terms of the number of hidden layers and the number of nodes: (**a**) with one hidden layer, (**b**) two hidden layers, and (**c**) three hidden layers.

To determine the appropriate number of hidden layers and the nodes used in each layer, we evaluated the estimation performance by varying the number of those hyper parameters. In addition, we estimated the training time of the deep neural network while varying the hyper parameters. Figure 9 shows the training time and the estimation accuracy of each different hyper parameter. For example, in the case of using one hidden layer, we increased the number of nodes in the hidden layer from 1 to 34, which is the same size as the input data, and marked the corresponding accuracy and training time for each case with blue dots. Even when two or three hidden layers were used, the number of nodes used for each hidden layer was set to a minimum of 1 and a maximum of 34. In other words, when two hidden layers are used, a total of 1156 (i.e., 34 × 34) structures are generated, and in the case of three hidden layers, a total of 39304 (i.e., 34 × 34 × 34) structures are generated.

**Figure 10:**
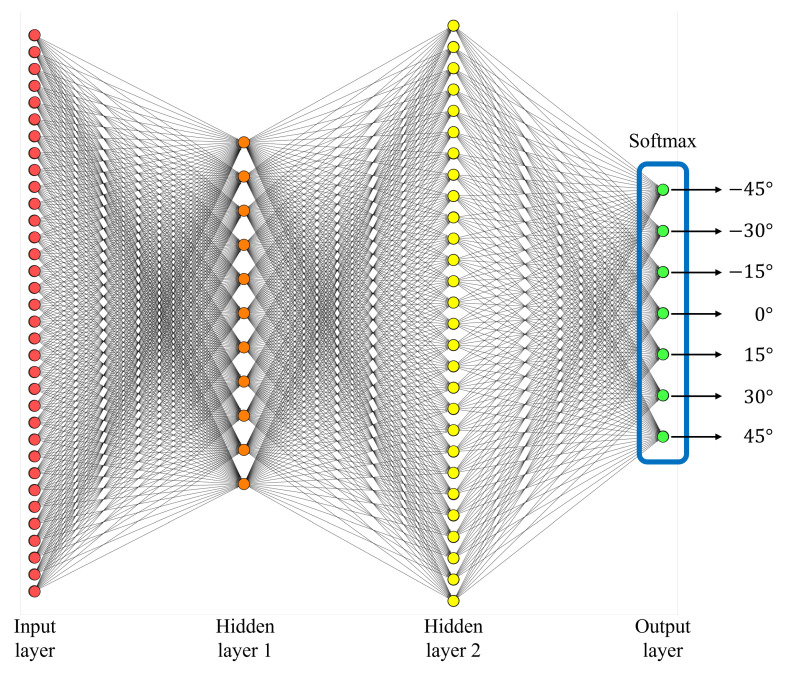
Proposed DNN structure for vertical tilt angle estimation considering accuracy and complexity.

In terms of training time, the case of using one hidden layer has an advantage over the case of using two hidden layers, but when only one hidden layer is used, the maximum accuracy is 90.83%, which is 8.25%p lower than the case of using two hidden layers. In addition, if three hidden layers are used, the maximum accuracy is only slightly higher than when using two hidden layers, with a difference of less than 1%p. However, the training time required for the network with three hidden layers increases by more than 10%, which may not be worth the marginal improvement in accuracy. Therefore, we decided to utilize the highest accuracy DNN classifier structure with two hidden layers to determine the vertical tilt angle of the automotive radar. The structure with the highest accuracy uses 11 nodes for the first hidden layer and 28 nodes for the second hidden layer, as shown in Figure 10.

### 4.3. Performance Evaluation

**Figure 11:**
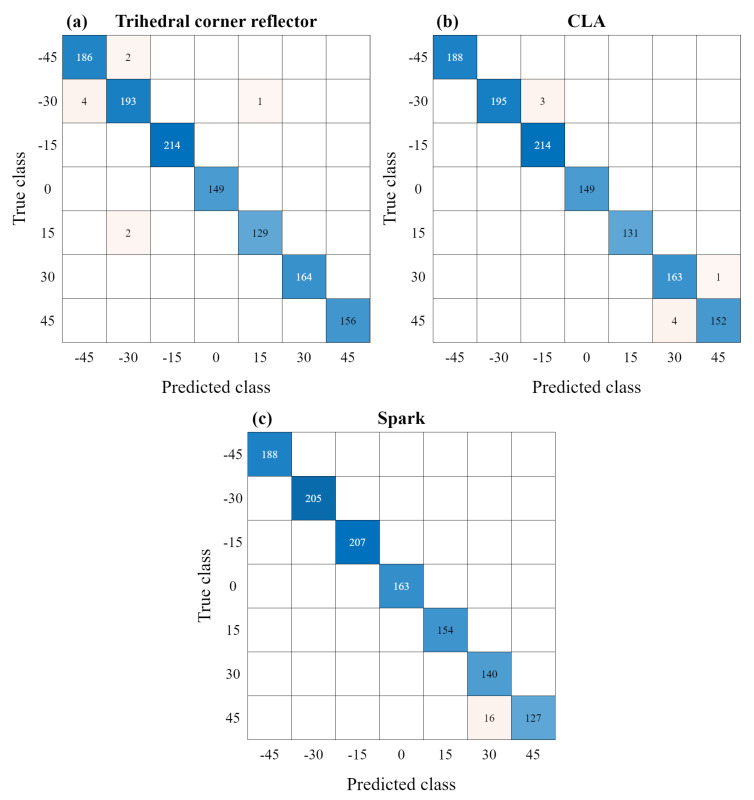
Confusion matrices for the tilt angle estimation using the proposed DNN-based classifier: (**a**) trihedral corner reflector, (**b**) CLA, and (**c**) Spark.

First, when a new radar signal is received, the FFT is applied to obtain the range profile in the frequency-domain. Then, an input vector of length 34 containing all signal components for the target is acquired and utilized as an input for the proposed DNN-based classifier, as shown in Figure 10. Finally, the proposed deep learning-based classifier generates a final estimate of one of the seven tilt angle values regardless of the measured distance. Table 3 shows the estimation performance of the proposed DNN classifier. For our test datasets, the estimation accuracies of the trihedral corner reflector, CLA, and Spark were 99.25%, 99.33%, and 98.67%, respectively. Overall, the proposed misalignment angle estimator showed an average estimation accuracy of 99.08%. In addition, Figure 11 shows the confusion matrices for the tilt angle estimation accuracy of each of the three targets. As shown in Figure 11, the accuracy of determining the initial mounting condition (i.e., $0^{\circ }$) and the misalignment condition is 100% for all targets.

**Figure 12:**
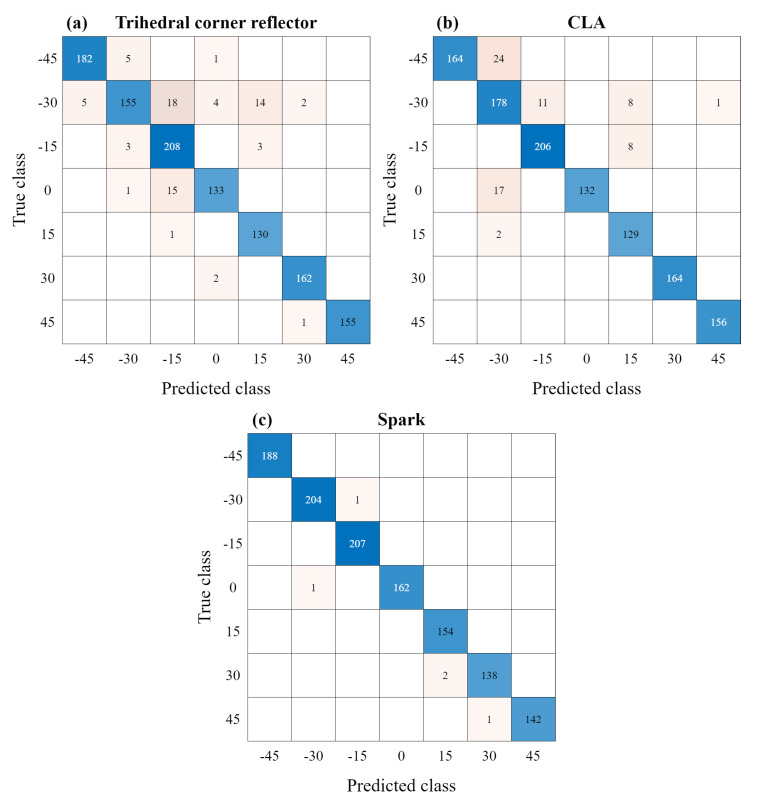
Confusion matrices for the tilt angle estimation using decision tree: (**a**) trihedral corner reflector, (**b**) CLA, and (**c**) Spark.

Moreover, the performance comparison was conducted using the decision tree [36], which is widely known as a feature-based machine learning algorithm for classification tasks. The same input vectors, as shown in Figure 8b, were used as input for the decision tree. The estimation results using the decision tree are shown in Figure 12, which are the confusion matrices using decision tree. In this case, the tilt angle estimation accuracy of each of the three targets is 93.8%, 94.1%, and 99.6%, respectively, showing an average estimation accuracy of 95.83%. In addition, the accuracy of distinguishing between the initial mounting state and the misalignment state is 98.25, 98.58%, and 99.92% for each target. Therefore, the proposed DNN-based estimation technique is more efficient than the feature-based machine learning method.

## 5. Conclusions

In this paper, we proposed an efficient method for estimating the vertical misalignment state of automotive radar system by using the DNN-based classifier. The signal received through the automotive FMCW radar was converted into a range profile and used as the input. Then, we extracted input vectors containing signal components between 0 and 4 m where the target exists and passes them through the proposed DNN. Finally, the DNN estimates the misalignment angle from the input vector. When evaluating the performance with the acquired radar datasets, the proposed DNN-based misalignment angle estimator classified the sensor data for 7 tilt angles with an average accuracy of 99.08% regardless of the measurement distance. In addition, our proposed method showed 100% of misalignment condition discrimination accuracy. Furthermore, we compared the estimation performance with other feature-based machine learning algorithm and confirmed that our proposed method is superior. This suggests that the proposed DNN-based estimator can be used to effectively detect the misalignment of the automotive radar system with a simple inspection that does not require a professional technician and avoids the need for disassembling the vehicle. However, as the proposed method uses data measured at 15 degree intervals, it is possible to determine the misalignment condition, but there is a limit to precisely estimating the radar mounting angle. Therefore, future work through data measured at smaller angular intervals should be conducted.

## Figures and Tables

**Table 1:** Specifications of the FMCW Radar Sensor Used in the Measurement.

System Parameter	Value
Center frequency, $f_{c}$	77 GHz
Bandwidth, *B*	3 GHz
Chirp duration, $T_{c}$	160 μs
The number of chirps, $N_{c}$	128
The number of time samples, *N*	256
Sampling frequency, $f_{s}$	10 MHz
Frame time, $T_{f}$	20.48 ms
Range resolution, $R_{res}$	0.1172 m
Maximum detectable range, $R_{max}$	29.9972 m
The number of transmit antenna element, $N_{T}$	1
The number of receiving antenna elements, $N_{R}$	4

**Table 2:** Summary of Measurement Scenarios.

Target	Measurement Distance	Tilt Angle
Trihedral corner reflector	1 m, 2 m, 3 m	$-45^{\circ },\;-30^{\circ },\;-15^{\circ },\;0^{\circ },$
		$15^{\circ },\;30^{\circ },\;45^{\circ }$
Mercedes-Benz CLA	1 m, 2 m, 3 m	$-45^{\circ },\;-30^{\circ },\;-15^{\circ },\;0^{\circ },$
		$15^{\circ },\;30^{\circ },\;45^{\circ }$
Chevrolet Spark	1 m, 2 m, 3 m	$-45^{\circ },\;-30^{\circ },\;-15^{\circ },\;0^{\circ },$
		$15^{\circ },\;30^{\circ },\;45^{\circ }$

**Table 3:** Radar Tilt Angle Estimation Performance of Each Target.

Target	Estimation Accuracy
Trihedral corner reflector	99.25%
Mercedes-Benz CLA	99.33%
Chevrolet Spark	98.67%

## Data Availability

Data sharing not applicable.

## Abbreviations

The following abbreviations are used in this manuscript:

ADC	Analog-to-digital converter
DNN	Deep neural network
DSP	Digital signal processor
FFT	Fast Fourier transform
FM	Frequency Mixer
FMCW	Frequency-modulated continuous wave
I	In-phase
LPF	Low pass filter
Q	Quadrature
Rx	Receiving antenna
Tx	Transmitting antenna
VCO	Voltage-controlled oscillator
WG	Waveform generator
